# Missions of the pre-hospital emergency system of Mazandaran following child drowning and related demographic risk factors

**Published:** 2023-12

**Authors:** Yahya Saleh Tabari, Zoya Hadinejad, Zeinab Sajadi, Mohammad Shadman, Neda Rahmatnejad, Maryam Mohseni, Hassan Talebi Ghadicolaei

**Affiliations:** ^*a* School of Medicine, Pharmaceutical Science Research Center, Hemoglobinopathy Institute, Mazandaran University of Medical Sciences, Sari, Iran. ^; ^*b* Health in Emergency and Disaster Research Center, University of Social Welfare and Rehabilitation Sciences, Tehran, Iran. ^; ^*c* Emergency Medical Services, Mazandaran University of Medical Sciences, Sari, Iran. ^

## Abstract

**Background:**

Drowning is a critical health problem worldwide, and the World Health Organization reported that about 360,000 people died due to drowning in 2015. More than half of these deaths are related to people under 25. Based on WHO, the highest rate of drowning deaths occurs among children aged 1 to 4 years, followed by children aged 5-9 years. This study aimed to determine the epidemiological factors as well as other factors affecting drowning in the age group under 18 years old in the pre-hospital emergency system of Mazandaran University of Medical Sciences, Iran.

**Methods:**

This descriptive cross-sectional study was conducted to investigate mission forms of all under 18-year-old ones with drowning in the EMS center of Mazandaran from April to July 2023. Items such as age, sex, place of drowning, the result of the mission, and the time of the accident were extracted.

**Results:**

Of 127 children who were rescued after drowning accident, 87 cases (68.5%) were male. The mean age of drowned children was 12 years. The highest rate of drowning was recorded for the age group of 11-18 years (n=86, (67.7%), followed by 19 victims in the age group of 6-8 years (15%). About 17 children (13.4%) died before the ambulance arrived, and 9 (7.1%) were transferred to the medical center during cardiopulmonary resuscitation. The highest number of drowning cases belonged to the group who were transferred to the medical centers (n=74, 58.3%). The mean age of the deceased was 9 years old.

**Conclusions:**

Based on the findings, over one-fifth of the drowned children died before the ambulance arrived or received advanced cardiopulmonary resuscitation. Addressing the determinants of health affecting drowning requires a multi-sectoral approach and inevitably a multi-sectoral action plan to prevent drowning in Iran. Training is a critical component of preventing the risk of drowning in children.

**Keywords:**

Children, Epidemiology, Drowning, Emergency Medical Services, Prevention

